# Determinants of arterial elastic function in middle-aged and elderly people: A population-based cross-sectional study from a low-income population in China

**DOI:** 10.3389/fcvm.2023.1037227

**Published:** 2023-02-09

**Authors:** Jiayi Sun, Zhen Zhang, Yunhan Fei, Yannan Gao, Zejian Li, Shuai Gao, Yunfan Wang, Jie Liu, Jun Tu, Haiying Wang, Jinghua Wang, Xianjia Ning, Wenjuan Zhao, Wenjuan Zhang

**Affiliations:** ^1^Department of Cardiology, Tianjin Medical University General Hospital, Tianjin, China; ^2^Department of Emergency, Tianjin Huanhu Hospital, Tianjin, China; ^3^Tianjin Key Laboratory of Cerebral Vascular and Neurodegenerative Diseases, Tianjin, China; ^4^School of Basic Medical Sciences, Tianjin Medical University, Tianjin, China; ^5^Department of Neurology, Tianjin Medical University General Hospital, Tianjin, China; ^6^Laboratory of Epidemiology, Tianjin Neurological Institute, Tianjin, China; ^7^Tianjin Neurological Institute, Key Laboratory of Post-Neuroinjury Neuro-Repair and Regeneration in Central Nervous System, Ministry of Education, Tianjin, China; ^8^Institute of Clinical Epidemiology and Evidence-Based Medicine, Tianjin Jizhou People’s Hospital, Tianjin, China; ^9^Department of Cardiology, Tianjin Jizhou People’s Hospital, Tianjin, China

**Keywords:** arterial elastic function, middle-aged and elderly people, risk factors, atherosclerosis, epidemiology

## Abstract

**Background:**

Arterial stiffness is closely associated with the occurrence of many cardiovascular and cerebrovascular diseases. However, the risk factors and mechanisms related to arterial stiffness development have only been partially elucidated. We aimed to describe arterial elastic function and its influencing factors in middle-aged and elderly people in rural China.

**Methods:**

This was a cross-sectional study conducted among residents, aged ≥45 years, of Tianjin, China, between April and July 2015. Data regarding participant demographics, medical history, lifestyle, and physical examination results were collected and assessed the association with arterial elastic function using linear regression.

**Results:**

Of the 3,519 participants, 1,457 were male (41.4%). Brachial artery distensibility (BAD) decreased by 0.5%/mmHg with every 10-year increment in age. The mean BAD value was 0.864%/mmHg lower in women than in men. With each unit increase in mean arterial pressure, the BAD decreased by 0.042%/mmHg. In patients with hypertension or diabetes, the BAD decreased by 0.726 and 0.183%/mmHg, respectively, compared with those without hypertension or diabetes. For each unit increase in triglyceride (TG) level, the mean BAD increased by 0.043%/mmHg. With each increase in body mass index (BMI) category, the BAD increased by 0.113%/mmHg. Brachial artery compliance (BAC) decreased by 0.007 ml/mmHg with each 10-year increase in age, and brachial artery resistance (BAR) increased by 30.237 dyn s^–1^ cm^–5^. The mean BAC in women was 0.036 ml/mmHg lower and the mean BAR was 155.231 dyn s^–1^ cm^–5^ higher in women than in men. In individuals with hypertension, the mean BAC decreased by 0.009 ml/mmHg and the mean BAR increased by 26.169 dyn s^–1^ cm^–5^. With each increase in BMI category, the mean BAC increased by 0.005 ml/mmHg and the mean BAR decreased by 31.345 dyn s^–1^ cm^–5^. For each unit increase in TG level, the mean BAC increased by 0.001 ml/mmHg.

**Conclusion:**

These findings indicate that age, sex, mean arterial pressure, BMI, diabetes, hypertension, and TG level are independently associated with the components of peripheral arterial elasticity. Understanding the factors influencing arterial stiffness is important for developing interventions to minimize arterial aging and cardiovascular and cerebrovascular diseases caused by arterial aging.

## Introduction

Arterial stiffness is an underlying and ongoing process of vascular aging and is a major risk factor for cardiovascular disease (CVD)-related morbidity and mortality. In 2019, the Global Burden of Disease, Injury, and Risk Factors study revealed that ischemic heart disease and stroke are the leading causes of death and disability, worldwide (1, 2). Moreover, in 2016, the World Health Organization announced that the number of deaths related to CVD was 17.9 million, accounting for 31% of all deaths in 2016, and is expected to reach 22.2 million by 2030 (3). The situation in China is even more severe (4). Therefore, focusing on the characteristics and risk factors of arterial stiffness in the Chinese population is important for preventing early progression of arterial stiffness.

Understanding the factors influencing arterial stiffness is particularly important for the management of CVDs in middle-aged and elderly people. Arterial elastic function can be used to indicate arterial stiffness, which has become an area of increased attention. Presently, studies in developed countries (e.g., the United States and the Netherlands) have found that sex may be a risk factor for arterial stiffness (5, 6). Similarly, a Chinese study showed that sex was a determinant of carotid artery stiffness, independent of age and blood pressure (BP) (7). Population-based findings have also shown that African-Americans have greater arterial stiffness than Whites (8). In the United Kingdom, results from a population-based, prospective study suggested that adequate control of pulse pressure (PP) and heart rate (HR), in conjunction with reduced inflammation, may delay aortic stiffness in men (9). However, some studies have shown that hypertensive patients with good long-term BP control have increased local arterial stiffness and endothelial dysfunction (10). In China, a study comparing prehypertensive populations showed that BP had a greater impact on vascular elastic function than blood lipid and sugar levels (11). These findings have limited implications for rural populations with low incomes and education levels, given the wide variations in socioeconomic status, environmental factors, and lifestyle patterns. The peripheral arterial elastic function characteristics and related influencing factors in the rural middle-aged and elderly individuals have not been well studied.

Therefore, in this population-based, cross-sectional study, we describe the functional characteristics of peripheral arterial elasticity and its associated factors in a low-income, middle-aged and elderly population, in rural China.

## Materials and methods

### Study population

This cross-sectional, population-based survey was conducted in rural areas of Tianjin, China. The study was performed between May and July 2015 among participants in the previously described Tianjin Brain Study (12). The study population consisted of 14,251 participants from 18 administrative villages in rural Tianjin. Approximately 95% of the participants were low-income farmers with an annual (2014) disposable income of less than $1600, per capita (13). All residents aged ≥45 years, without CVD, were recruited for this study; those with a history of CVD were excluded. Patients with other vascular diseases events, including acute coronary events, any form of coronary artery disease, or peripheral artery disease were also excluded. CVD and other vascular events were determined based on the patient’s medical history and imaging data.

All investigations were approved by the ethics committee of the Tianjin Medical University General Hospital. The study was conducted in accordance with approved guidelines; informed consent was obtained from each participant.

### Information collection

All information in this study was collected through face-to-face interviews conducted by trained researchers using predesigned questionnaires. To examine the consistency of investigator visits, 50 participants were recruited in advance for this study and interviewed by 5 investigators each. The Kendall’s *W* test was used in this study to analyze the agreement in questionnaire assesment among the five investigators, and the results showed a strong agreement with a Kendall’s *W* coefficient of 0.967, *P* < 0.001.

Information on demography, previous disease history, individual life style, physical examination results, and laboratory indictors was included in the questionnaires. The demographical information included sex, and age; previous disease history included hypertension and diabetes; information on individual life style included smoking and drinking habits. Moreover, height, weight, BP, HRs, arterial elastic function [including brachial artery compliance (BAC), brachial artery distensibility (BAD), and brachial artery resistance (BAR), and laboratory indictors [including fasting blood glucose, total cholesterol (TC), triglycerides (TG), high-density lipoprotein cholesterol (HDL-C), low-density lipoprotein cholesterol (LDL-C)] were obtained in this study.

### Measurement of arterial elasticity function parameters

Participants rested quietly for 5 min and measurements were taken using DynaPulse (Pulse Metric, San Diego, CA, USA), a non-invasive BP and hemodynamic monitoring device. During the measurements, the instrument automatically recorded the patient’s BP and brachial artery pulse waveform (PWF); the instrument’s software then calculated the indicators of arterial elastic function: BAC, BAD, and BAR.

### Physical examinations and blood tests

Blood pressure measurements were taken with the participant sitting in a quiet room of the local village health center. All participants were advised to avoid smoking and consuming alcohol, tea, or coffee before having their BP measured, per the American Hypertension Association criteria, using an electronic sphygmomanometer. Before the measurement, each participant rested in a quiet room for at least 5 min. Then, the sphygmomanometer cuff was placed on the upper arm at the same level as the heart, and the cuff tightness was adjusted according to the participant’s arm circumference. Each participant’s SBP, DBP, and HR was recorded. PP was defined as the difference between SBP and DBP; mean arterial pressure (MAP) was calculated as (SBP + 2 × DBP) / 3. Each participant’s height and weight was measured in their local village health center clinic, and the body mass index (BMI) was calculated as weight (kg)/height (m)^2^.

Venous blood samples were collected and sent, within 2 h of collection, to the central laboratory at Tianjin Jizhou People’s Hospital for the measurement of TC, TG, HDL-C, and LDL-C levels using standardized enzymatic methods.

### Grouping and risk factor definitions

Personal lifestyle characteristics included smoking and drinking habits. Smoking was defined as smoking >1 cigarette per day for at least 1 year; participants were divided into the smoking and non-smoking groups according to their current smoking status. Alcohol consumption was defined as drinking more than 500 g per week for at least 1 year, and participants were divided into the drinking and non-drinking groups, according to their reported alcohol consumption.

Hypertension was defined as systolic BP (SBP) ≥140 mmHg, diastolic BP (DBP) ≥90 mmHg, or the use of antihypertensive medication (14). Diabetes was defined as a fasting blood glucose level ≥7.0 mmol/L or taking oral hypoglycemic agents or insulin (15). The diagnostic criteria for hyperlipidemia were TC ≥6.2 mmol/L, low-density lipoprotein cholesterol LDL-C ≥4.1 mmol/L, HDL-C <1.0 mmol/L, and TG ≥2.3 mmol/L; if any one of these criteria were met, the patient was diagnosed as having hyperlipidemia (16).

Participants were divided into four age groups: 45–54, 55–64, 65–74, ≥75 years. According to specific Asian population criteria, the participants were divided into normal (18.5 ≤ BMI < 24 kg/m^2^), overweight (24 ≤ BMI < 28 kg/m^2^), and obese (BMI ≥ 28 kg/m^2^) groups (17).

### Statistical analyses

Forest plots were constructed for subgroup analyses using Stata/MP 16 (StataCorp, College Station, TX, USA). Continuous variables (age, laboratory parameters, BP components, and peripheral arterial elasticity parameters) are expressed as means with standard deviations; Student’s *t*-tests or analyses of variance were used to compare differences between two or multiple groups, respectively. Categorical variables (smoking, alcohol consumption, hypertension, diabetes, and hyperlipidemia) are represented as numbers and frequencies; between group comparisons were made using the Chi-square test. Variables with *P* < 0.05 in the univariate analysis were included in the multiple linear regression, and the factors related to arterial elasticity parameters were analyzed using linear regression; statistical significance was set at *P* < 0.05. Statistical analyses were performed using for statistical version 25.0 (SPSS, Chicago, IL, USA).

## Results

### Demographic characteristics of participants

The average age of the 3,519 participants (2,062 women, 58.6%) was 61.50 years, including 1,291 (36.7%) who were aged 55–64 years; the average age of the male participants was 62.82 years and that of the female participants was 60.54 years. The prevalences of hypertension, diabetes, and hyperlipidemia in this study population were 73.5, 12.1, and 28.7%, respectively. The mean SBP (150.73 mmHg), DBP (85.59 mmHg), MAP (107.15 mmHg), and PP (65.14 mmHg) values were higher than desirable levels. The average BMI of this population was 25.70 kg/m^2^, with 76.8% of the participants having a BMI of <28 kg/m^2^. Among the participants, 19.8% smoked and 13.0% drank (Table 1).

**TABLE 1:** Baseline characteristics of male and female.

Characteristics	Male	Female	Total
Cases, *n* (%)	1,457 (41.4)	2,062 (58.6)	3,519 (100)
Age, year, mean (SD)	62.82 (9.66)	60.54 (9.15)	61.50 (9.44)
**Age group, *n* (%)**			
45–54 years	345 (23.7)	624 (30.7)	979 (27.8)
55–64 years	510 (35.0)	781 (37.9)	1,291 (36.7)
65–74 years	425 (29.2)	500 (24.2)	925 (26.3)
≥75 years	177 (12.1)	147 (7.1)	324 (9.2)
**Risk factors, *n* (%)**			
Hypertension	1,110 (76.2)	1,478 (71.7)	2,588 (73.5)
Diabetes mellitus	158 (10.8)	269 (13.0)	427 (12.1)
Hyperlipidemia	398 (27.3)	612 (29.7)	1,010 (28.7)
Smoking	652 (44.7)	45 (2.2)	697 (19.8)
Drinking	435 (29.9)	21 (1.0)	456 (13.0)
**Laboratory tests, mean (SD)**			
TC, g/L	4.71 (0.89)	5.06 (0.95)	4.92 (0.94)
TG, g/L	1.62 (2.55)	1.73 (1.60)	1.69 (2.05)
HDL-C, g/L	1.34 (0.34)	1.40 (0.49)	1.38 (0.43)
LDL-C, g/L	3.09 (6.76)	3.14 (5.22)	3.12 (5.90)
BMI (kg/m^2^)	25.23 (3.49)	26.03 (8.52)	25.70 (6.91)
**BMI group, *n* (%)**			
Normal	520 (36.3)	620 (30.6)	1,140 (32.9)
Overweight	620 (43.2)	880 (43.4)	1,500 (43.4)
Obese	294 (20.5)	526 (26.0)	820 (23.7)
Systolic BP (mmHg)	150.91 (22.10)	150.61 (23.62)	150.73 (23.00)
Diastolic BP (mmHg)	87.99 (11.11)	83.90 (11.13)	85.59 (18.47)
MAP (mmHg)	108.81 (13.84)	105.97 (14.35)	107.15 (14.20)
PP (mmHg)	62.92 (17.69)	66.71 (18.85)	65.14 (18.47)
HR (bpm)	71.92 (11.19)	73.88 (10.54)	73.07 (10.86)
LV CO (L/min)	4.86 (0.93)	4.84 (0.96)	4.85 (0.95)
BAD (%/mmHg)	5.67 (1.57)	5.09 (1.49)	5.33 (1.55)
BAC (ml/mmHg)	0.08 (0.02)	0.05 (0.01)	0.06 (0.02)
BAR (dyn⋅s^–^ cm^–5^)	144.82 (75.24)	289.24 (139.12)	229.23 (136.85)

BMI, body mass index; BP, blood pressure; MAP, mean arterial pressure; PP, pulse pressure; HR, heart rate; CO, cardiac output; BAC, brachial artery compliance; BAD, brachial artery distensibility; BAR, brachial artery resistance; TC, total cholesterol; LDL-C, low-density lipoprotein cholesterol; HDL-C, high-density lipoprotein cholesterol; TG, triglycerides.

The mean left ventricular output (LV CO) in this study population was 4.85 L/min; the mean BAR was 229.23 ± 136.85 dyn s^–1^ cm^–5^. The mean BAD value in men was 5.67 ± 1.57%/mmHg, whereas that in women was 5.09 ± 1.49%/mmHg; men had a mean BAC value of 0.08 ± 0.02 ml/mmHg and women of 0.05 ± 0.01 ml/mmHg (Table 1).

### Arterial elastic function following age stratification

The average BAD value was the largest in the youngest age group (6.00 ± 1.47%/mmHg). With increasing age, the average BAD gradually decreased; the lowest value was 4.22 ± 1.27%/mmHg in the ≥75-year-old group (*P* < 0.001). The mean BAC value in the 45–54-year-old group was the highest among the four participant age groups (0.07 ± 0.03 ml/mmHg, *P* < 0.001). With increasing age, the average BAR values for the various age groups gradually increased, with the ≥75-year-old group having the largest mean value (288.84 ± 186.96 dyn s^–1^ cm^–5^, *P* < 0.001) (Table 2).

**TABLE 2:** Comparison of arterial elasticity components in different age groups.

	45–54 years	55–64 years	65–74 years	≥75 years	*P*
BAD (%/mmHg)	6.00 (1.47)	5.40 (1.50)	4.89 (1.42)	4.22 (1.29)	<0.001
BAC (ml/mmHg)	0.07 (0.03)	0.06 (0.02)	0.06 (0.02)	0.05 (0.02)	<0.001
BAR (dyn s^–^ cm^–5^)	198.19 (109.52)	225.33 (128.51)	247.19 (143.76)	288.84 (186.96)	<0.001

### Univariate analysis of functional components of arterial elasticity

In the linear regression analysis the BAD, BAC, and BAR values were used as dependent variables; age, SBP, DBP, MAP, PP, HR, TG level, TC level, LDL-C level, HDL-C level, LV CO, BMI, smoking, drinking, hypertension, and diabetes were used as independent variables.

Our results showed that unadjusted age, sex, SBP, DBP, MAP, PP, TG, TC, LDL-C, LV CO, BMI, hypertension, and diabetes were negatively associated with the BAD value (*P* < 0.05); HR, smoking, and alcohol consumption were positively correlated with the BAD value (*P* < 0.05). Unadjusted age, sex, PP, TC, LDL-C, HDL-C, hypertension, and diabetes were negatively correlated with the BAC value (*P* < 0.05), whereas DBP, LV CO, BMI, smoking, and drinking were positively correlated with the BAC value (*P* < 0.05). Unadjusted age, sex, SBP, PP, TC, LDL-C, HDL-C, and hypertension were positively correlated with the BAR value (*P* < 0.05), whereas DBP, HR, LV CO, BMI, smoking, and drinking were negatively correlated with the BAR value (*P* < 0.05) (Table 3).

**TABLE 3:** Univariate analysis of arterial elastic components.

	BAD (%/mmHg)	BAC (ml/mmHg)	BAR (dyn s^–^ cm^–5^)
AGE group	−0.575 (−0.629, −0.521)*	−0.007 (−0.008, −0.006)*	27.012 (22.805, 32.418)*
Sex	−0.580 (−0.687, −0.472)*	−0.034 (−0.035, −0.032)*	144.412 (136.445, 152.379)*
SBP	−0.050 (−0.052, −0.049)*	–	1.523 (1.331, 1.715)*
DBP	−0.012 (−0.017, −0.007)*	0.001 (0.001, 0.001)*	−3.710 (−4.097, −3.324)*
MAP	−0.054 (−0.057, −0.050)*	–	–
PP	−0.074 (−0.075, −0.073)*	−0.001(−0.001, −0.001)*	3.728 (3.515, 3.941)*
HR	0.021 (0.016, 0.026)*	–	−4.027 (−4.430, −3.625)*
TG quartiles group	−0.070 (−0.119, −0.022)*	–	–
TC quartiles group	−0.122 (−0.171, −0.074)*	−0.003 (−0.004, −0.002)*	8.843 (4.766, 12.921)*
LDL-C quartiles group	−0.098 (−0.147, −0.050)*	−0.002 (−0.002, −0.001)*	4.631 (0.534, 8.727)*
HDL-C quartiles group	–	−0.003 (−0.004, −0.002)*	13.405 (9.316, 17.494)*
LV CO	−0.277 (−0.333, −0.222)*	0.001 (0, 0.002)*	−39.891 (−44.338, −35.445)*
BMI group	−0.049 (−0.122, −0.023)*	0.004 (0.003, 0.005)*	−27.214 (−33.252, −21.177)*
Smoking	0.223 (0.109, 0.338)*	0.014 (0.012, 0.016)*	−56.815 (−66.460, −47.170)*
Drinking	0.288 (0.147, 0.429)*	0.019 (0.017, 0.021)*	−76.832 (−88.503, −65.161)*
Hypertension	−1.635 (−1.743, −1.527)*	−0.008 (−0.010, −0.006)*	26.801 (16.401, 37.201)*
Diabetes	−0.433 (−0.597, −0.270)*	−0.004 (−0.007, −0.002)*	–

*P < 0.05.

### Multivariate analysis of arterial elastic components

Using BAD, BAC, and BAR as dependent variables, the variables with the *P*-values < 0.05 in the univariate analysis were included in the multiple linear regression (Table 4). Since there is collinearity among the SBP, DBP, MAP, PP, HR, and LV CO variables, and because TC, TG, HDL-C, and LDL-C have a linear relationship, we selected only MAP, LDL-C, and TG as variables of interest.

**TABLE 4:** Multivariate analysis of arterial elasticity parameters.

	BAD (%/mmHg)	BAC (ml/mmHg)	BAR (dyn s^–^ cm^–5^)
AGE group	−0.500 (−0.547, −0.453)*	−0.007 (−0.008, −0.007)*	30.237 (26.215, 34.258)*
Sex	−0.864 (−0.965, −0.764)*	−0.036 (−0.038, −0.035)*	155.231 (146.704, 163.758)*
MAP	−0.042 (−0.046, −0.038)*	–	–
TG quartiles group	0.043 (0.002, 0.083)*	0.001 (0.000, 0.002)*	–
LDL-C quartiles group	–	–	–
BMI group	0.113 (0.052, 0.173)*	0.005 (0.005, 0.006)*	−31.345 (−36.364, −26.327)*
Smoking	–	–	–
Drinking	–	–	–
Hypertension	−0.726 (−0.850, −0.601)*	−0.009 (−0.011, −0.008)*	26.169 (15.626, 36.713)*
Diabetes	−0.183 (−0.312, −0.055)*	–	–

*P < 0.05.

**FIGURE 1:**
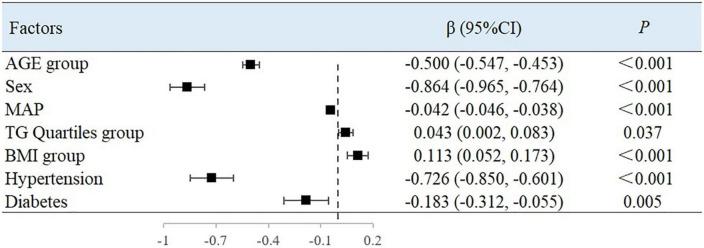
Factors associated with BAD in multivariate analysis.

The results showed that with every increase in the examined 10-year age categories, the mean BAD value decreased by 0.5%/mmHg [95% confidence interval (CI), −0.547 to −0.453; *P* < 0.001). Compared with men, the mean BAD value was 0.864%/mmHg lower in women (95% CI, −0.965 to −0.764; *P* < 0.001). With each unit increase in MAP, the BAD value decreased by 0.042%/mmHg (95% CI, −0.046 to −0.038; *P* < 0.001). Compared with people without hypertension or diabetes, the BAD value decreased by 0.726%/mmHg (95% CI, −0.850 to −0.601; *P* < 0.001) and 0.183%/mmHg (95% CI, −0.312 to −0.055; *P* = 0.005) among individuals with hypertension or diabetes, respectively. For each unit of TG, the BAD value increased by 0.043%/mmHg (95% CI, 0.002–0.083; *P* = 0.037). Between each of the BMI groupings used in this study, the BAD value increased by 0.113%/mmHg (95% CI, 0.052–0.173; *P* < 0.001) (Figure 1).

**FIGURE 2:**
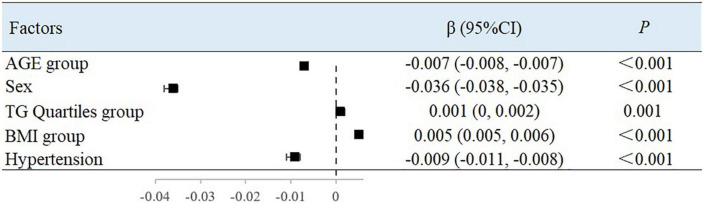
Factors associated with BAC in multivariate analysis.

**FIGURE 3:**
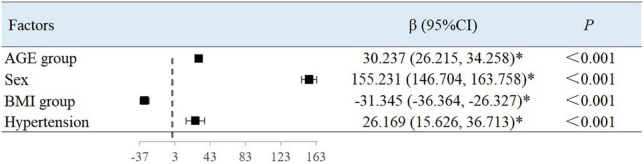
Factors associated with BAR in multivariate analysis.

After adjusting for variables, with each 10-year increase in age grouping used in the study, the BAC value decreased by 0.007 ml/mmHg (95% CI, −0.008 to −0.007; *P* < 0.001) (Figure 2) and the BAR value increased by 30.237 dyn s^–1^ cm^–5^ ((95% CI, 26.215–34.258; *P* < 0.001) (Figure 3). Women had a mean BAC value that was 0.036 ml/mmHg (95% CI, −0.038 to −0.035; *P* < 0.001) lower and a mean BAR value that was 155.231 dyn s^–1^ cm^–5^ (95% CI, 146.704–163.758, *P* < 0.001) higher than in men. Among participants with hypertension, the mean BAC value was 0.009 ml/mmHg (95% CI, −0.011 to −0.008; *P* < 0.001) lower and the mean BAR value was 26.169 dyn s^–1^ cm^–5^ (95% CI, 15.626–36.713; *P* < 0.001) higher than in those without hypertension. For each BMI category (normal to overweight to obese), the BAC value increased by 0.005 ml/mmHg (95% CI, 0.005–0.006; *P* < 0.001) and the BAR value decreased by 31.345 dyn s^–1^ cm^–5^ (95% CI, −36.364 to −26.327; *P* < 0.001), relative to the next lower BMI group. For each unit increase in TG level, the BAC value increased by 0.001 ml/mmHg (95% CI, 0.000–0.002; *P* = 0.001).

### Associated factors in the subgroup analyses stratified by age

**FIGURE 4:**
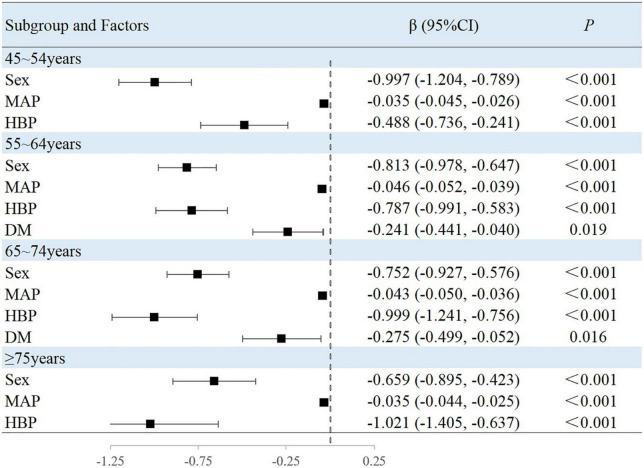
Factors associated with BAD in subgroup analysis by age.

**FIGURE 5:**
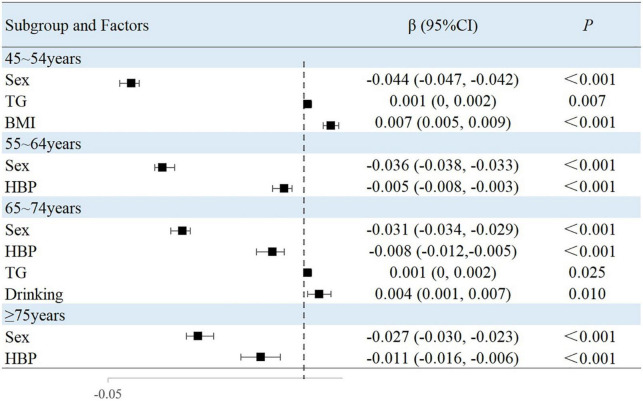
Factors associated with BAC in subgroup analysis by age.

**FIGURE 6:**
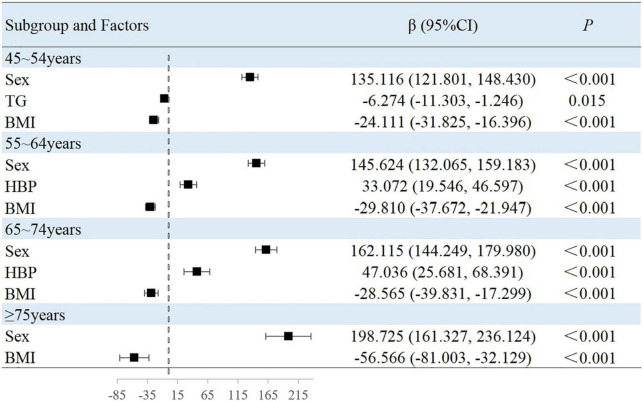
Factors associated with BAR in subgroup analysis by age.

The results of this study showed that sex, MAP, history of hypertension, TG level, and BMI were independently associated with peripheral arterial elasticity parameters in people aged 45–54 years. In this age group, the mean BAD value was 0.997%/mmHg lower (95% CI, −1.204 to −0.789; *P* < 0.001) (Figure 4), mean BAC value was 0.044 ml/mmHg lower (95% CI, −0.047 to −0.042; *P* < 0.001) (Figure 5), and mean BAR value was 135.116 dyn s^–1^ cm^–5^ higher (95% CI, 121.801–148.430; *P* < 0.001) in women than in men (Figure 6). For each unit increase in MAP, the mean BAD value decreased by 0.035%/mmHg (95% CI, −0.045 to −0.026; *P* < 0.001). In individuals with hypertension, the mean BAD value was 0.488%/mmHg lower (95% CI, −0.736 to −0.241; *P* < 0.001) than in those without hypertension.

For each unit increase in TG, the mean BAC value increased by 0.001 ml/mmHg (95% CI, 0.000–0.002; *P* = 0.007) and the mean BAR value decreased by 6.274 dyn s^–1^cm^–5^ (95% CI, −11.303 to −1.246; *P* = 0.015). Moreover, each increase in BMI category resulted in the mean BAC value increasing by 0.007 ml/mmHg (95% CI, 0.005–0.009; *P* < 0.001) and the mean BAR value decreasing by 24.111 dyn s^–1^ cm^–5^ (95% CI, −31.825 to −16.396; *P* < 0.001).

In the 55–64-year-old subgroup, the mean BAD value was 0.813%/mmHg (95% CI, −0.978 to −0.647; *P* < 0.001) lower, the mean BAC value was 0.036 ml/mmHg (95% CI, −0.038 to −0.033; *P* < 0.001) lower, and the mean BAR value was 145.624 dyn s^–1^ cm^–5^ (95% CI, 132.065–159.183; *P* < 0.001) higher in women than in men. In this subgroup, with every unit increase of the mean MAP, the BAD value decreased by 0.046%/mmHg (95% CI, −0.052 to −0.039; *P* < 0.001). Among those with hypertension, the mean BAD value decreased by 0.787%/mmHg (95% CI, −0.991 to −0.583; *P* < 0.001), the mean BAC value decreased by 0.005 ml/mmHg (95% CI, −0.008 to −0.003; *P* < 0.001), and the mean BAR value increased by 33.072 dyn s^–1^ cm^–5^ (95% CI, 19.546–46.597; *P* < 0.001) compared with those without hypertension. Among those with diabetes, the mean BAD value decreased by 0.241%/mmHg (95% CI, −0.441 to −0.040; *P* = 0.019) compared with those without diabetes. With the each increase in the BMI group, the mean BAR value decreased by 29.810 dyn s^–1^ cm^–5^ (95% CI, −37.672 to −21.947; *P* < 0.001).

In the 65–74-year-old subgroup, compared with men, women demonstrated a mean BAD decrease of 0.752%/mmHg (95% CI, −0.927 to −0.576; *P* < 0.001), a mean BAC decrease of 0.031 ml/mmHg (95% CI, −0.034 to −0.029; *P* < 0.001), and a mean BAR increase of 162.115 dyn s^–1^ cm^–5^ (95% CI, 144.249–179.980; *P* < 0.001). Among participants with hypertension, the mean BAD value decreased by 0.999%/mmHg (95% CI, −1.241 to −0.756; *P* < 0.001), the mean BAC value decreased by 0.008 ml/mmHg (95% CI, −0.012 to −0.005; *P* < 0.001), and the mean BAR value increased by 47.036 dyn s^–1^ cm^–5^ (95% CI, 25.681–68.391; *P* < 0.001). Among participants with diabetes, the mean BAD value decreased by 0.275%/mmHg (95% CI, −0.499 to −0.052; *P* = 0.016) compared with those without diabetes. Moreover, the mean BAC value of drinkers increased by 0.004 ml/mmHg (95% CI, 0.001–0.007; *P* = 0.010) compared with non-drinkers. With every increase in the BMI level grouping, the mean BAR value decreased by 28.565 dyn s^–1^ cm^–5^ (95% CI, −39.831 to −17.299; *P* < 0.001).

In the subgroup aged ≥75 years, women demonstrated a mean BAD value that was 0.659%/mmHg (95% CI, −0.895 to −0.423; *P* < 0.001) lower, a mean BAC value that was 0.027 ml/mmHg (95% CI, −0.030 to −0.023; *P* < 0.001) lower, and a mean BAR value that was 198.725 dyn s^–1^ cm^–5^ (95% CI, 161.327–236.124; *P* < 0.001) higher than in men. Among individuals with hypertension, the mean BAD value was 1.021%/mmHg (95% CI, −1.405 to −0.637; *P* < 0.001) lower and the mean BAC value was 0.011 ml/mmHg (95% CI, −0.016 to −0.006; *P* < 0.001) lower than in normotensive individuals. With each increase in the BMI level grouping, the mean BAR value decreased by 56.566 dyn s^–1^ cm^–5^ (95% CI, −81.003 to −32.129; *P* < 0.001).

## Discussion

This is the first study to describe the functional characteristics of arterial elasticity and its influencing factors in a middle-aged and elderly population in rural China. We found that age, female sex, MAP, hypertension, and diabetes were inversely associated, whereas TG level and BMI were positively associated with changes in BAD. We also found that increasing age, female sex, and a history of hypertension decreased BAC and increased BAR, whereas TG and BMI levels were positively associated with increases in BAC. In addition, BMI was negatively correlated with BAR. In an age-stratified subgroup analysis, female sex, MAP, hypertension, and diabetes were negatively correlated with changes in BAD; female sex and hypertension were also negatively correlated with changes in BAC, but positively correlated with changes in BAR. TG and BMI were positively correlated with BAC and negatively correlated with BAR.

As early as the 1920s, Bramwell and Hill (18) first reported that arterial stiffness measured using pulse wave velocity (PWV) was age-related; since then, increased arterial stiffness has since been considered to be a marker of early vascular aging. The results of recent studies on arterial stiffness have shown that age is the main risk factor for arterial stiffness, followed by BP (19). However, a cohort study showed that neither PP nor PWV increased with age, suggesting that arterial stiffness levels may be stable in healthy young adults with a low cardiovascular risk (20). Conversely, our results showed that age was related to arterial elastic function, with the average value of each arterial elastic function component differing between the age groups. Essentially, the results demonstrated that arterial dilatation and compliance gradually decreased with advancing age. The results of the multivariate linear regression analysis showed that with increasing age, the vascular compliance and dilatation decreased and the resistance increased, suggesting that the arterial elastic function weakened and arterial stiffness increased. Fatigue fractures of elastic elements are often considered to be responsible for age-related increases in aortic PWV (21). The results of a cross-sectional study showed that women had lower systemic vascular compliance and higher systemic vascular resistance than men (22). A longitudinal study showed that resistance increased in women and decreased in men with age (23). Consistently, the results of the present study showed that women had lower vascular compliance and distensibility, and increased resistance than men, even in the age subgroup analyses. Vascular aging is driven by oxidative stress, which can reduce the bioavailability of nitric oxide (NO) and stimulate changes in the extracellular matrix. These sex differences in vascular aging are attributed to changes in sex hormones that occur with age. Some studies have shown that testosterone can regulate vascular aging by reducing the effects of oxidative stress and inflammation (24). In women, the decrease in circulating estrogen during menopause accompanied by the aging process induces vascular dysfunction. Some studies have pointed out that regular aerobic exercise also enhances the endothelial function in men (by reducing oxidative stress and maintaining NO bioavailability) with increasing age, although this is inconsistent in postmenopausal women with estrogen deficiency (25).

An increase in MAP may accelerate arterial damage. Similarly, hypertension accelerates vascular aging and promotes the degradation of elastic fibers in the arterial wall; therefore, arteriosclerosis is also considered to be the result of long-term hypertension (26, 27). A population-based study showed that age and BP had a strong effect on arteriosclerosis (28). One study measured arterial stiffness and BP at different time points, longitudinally demonstrating the temporal relationship between arterial stiffness and BP increase; the onset of hypertension is associated with arterial stiffness (29, 30). Interestingly, two large studies arrived at different conclusions, showing that the progression of arterial stiffness was independent of initial BP levels (29, 31). These results suggest that arterial stiffness may precede the pathogenesis of hypertension. Our study describes the relationship between BP components and arterial elastic function, suggesting that increased MAP reduces vasodilatability and effects increased arterial stiffness. One of the possible reasons for the current controversies regarding the BP and arterial elasticity findings is that the BP measurements may be inaccurate, especially in older or cooperating individuals. Studies have indicated that the rate of elastin fragmentation may depend on the number of stress cycles and stress level, i.e., the HR and PP (32).

One cross-sectional study showed that in young adults, metabolic syndrome was associated with higher brachial-ankle PWV (33). The HR appears to have a dual effect on arterial stiffness, causing fatigue of the wall vessels with each heartbeat, while the HR can also increase vessel stiffness through the viscoelastic effects on wall composition (34). Research has suggested that control of body weight and cardiovascular risk factors is a potential target for interventions aimed at reducing the risk of developing arterial stiffness (35). Schmidt et al. showed that the weight gain commonly observed after smoking cessation was associated with an increase in PWV (36). Moderate to vigorous physical activity is associated with slower PWV progression, independent of other risk factors (37). The results of a follow-up study showed that weight loss improved PWV in as little as four weeks, and that dietary carbohydrate restriction may be an effective treatment for reducing aortic stiffness in women (38). These studies emphasized the importance of weight control in maintaining good arterial elastic function. The results of our study showed that being overweight or obese positively correlated with BAC and BAD, and negatively correlated with arterial resistance. This result differs from the results described in the aforementioned research. We are unable to explain the reason for this result, and further mechanistic research may be needed to clarify this.

A cohort study showed that chronic hyperglycemia adversely affected aortic stiffness in patients with Type 1 diabetes (39). Our results also consistently showed that patients with diabetes had reduced brachial artery vasodilatability, suggesting an increase in arterial stiffness. Indeed, arterial calcification, in turn, is related to vascular stiffening and arteriosclerosis. Data have shown that some mediators of bone mineralization are also regulators of arterial smooth muscle cell osteogenic transformation and arterial calcification in patients with diabetes (40). These observations suggest that osteoprotegerin may be directly involved in extraosseous calcification, resulting in stiffening of arteries and subsequent vascular disease in patients with Type 1 diabetes (41). The results of a cross-sectional study from China showed that SBP, DBP, PP, and MAP levels were positively correlated with brachial-ankle PWV (42). Our present findings showed that hypertension increased BAR and reduced BAD and BAC values; long-term hypertension increased arterial stiffness.

A recent, large cross-sectional study showed that TC levels were positively associated with PWV and that almost half of the increase in SBP in hypercholesterolemic individuals was mediated by arterial stiffness (43). A 5-year follow-up study showed that fluvastatin improved arterial stiffness in patients with coronary artery disease and hyperlipidemia (44). However, the results of this study showed that TG levels were positively correlated with both BAD and BAC values. Similarly, in our previous study, TGs were shown to prevent early atherosclerosis, after adjusting for underlying risk factors (45). We cannot explain the reason for this result, and more research is needed to clarify the mechanism.

## Limitations

Our study has some limitations. First, this was a population-based, cross-sectional study that limited our ability to clarify a causal relationship between the arterial elasticity parameters and their influencing factors. Second, as data on medical history were collected from existing data records or self-reported by participants, an unavoidable information bias may exist. In addition, we studied smoking and drinking indicators, defined as current drinking/smoking versus no drinking/smoking. However, exposure quantification methods were not used to adequately summarize an individual’s smoking and drinking history, which may have implications for the analysis of factors influencing arterial elastic function. In view of the limitations of this study, we hope to continue to improve data collection on factors that may influence arterial elasticity, continue to monitor arterial elasticity in populations, and plan prospective studies based on populations with low income and education level.

## Conclusion

In this study, age, sex, MAP value, BMI, diabetes, hypertension, and TG levels were independently associated with components peripheral arterial elasticity. A better understanding of the factors influencing arterial stiffness will help guide strategies for minimizing arterial aging and the cardiovascular and cerebrovascular diseases caused by arterial aging. To date, the risk factors and mechanisms of arterial stiffness have only been partially elucidated; further studies are needed to confirm these observations.

## Data availability statement

The raw data supporting the conclusions of this article will be made available by the authors, without undue reservation.

## Ethics statement

The studies involving human participants were reviewed and approved by the Ethics Committee of the Tianjin Medical University General Hospital. The patients/participants provided their written informed consent to participate in this study.

## Author contributions

WZhang, XN, and WZhao were involved in conception and design and critical review in for this article, and data interpretation for this article. JS, ZZ, YF, YG, ZL, SG, YW, JL, JT, and HW were involved in data collection, case diagnosis and confirmation for this article. JS, ZZ, and YF were involved in manuscript drafting. JW was involved in data analysis for this article. All authors contributed to the article and approved the submitted version.
